# Microbial Eukaryotes: a Missing Link in Gut Microbiome Studies

**DOI:** 10.1128/mSystems.00201-17

**Published:** 2018-03-13

**Authors:** Isabelle Laforest-Lapointe, Marie-Claire Arrieta

**Affiliations:** a Department of Physiology and Pharmacology, University of Calgary, Calgary, Alberta, Canada; b Department of Pediatrics, University of Calgary, Calgary, Alberta, Canada

**Keywords:** bacteria, *Blastocystis*, eukaryotes, fungi, gut microbiome, immune system, interkingdom interactions, microbial ecology, prokaryotes, protozoan

## Abstract

Human-associated microbial communities include prokaryotic and eukaryotic organisms across high-level clades of the tree of life. While advances in high-throughput sequencing technology allow for the study of diverse lineages, the vast majority of studies are limited to bacteria, and very little is known on how eukaryote microbes fit in the overall microbial ecology of the human gut. As recent studies consider eukaryotes in their surveys, it is becoming increasingly clear that eukaryotes play important ecological roles in the microbiome as well as in host health. In this perspective, we discuss new evidence on eukaryotes as fundamental species of the human gut and emphasize that future microbiome studies should characterize the multitrophic interactions between microeukaryotes, other microorganisms, and the host.

## Abstract (precis)

Human-associated microbial communities include prokaryotic and eukaryotic organisms across high-level clades of the tree of life. While advances in high-throughput sequencing technology allow for the study of diverse lineages, the vast majority of studies are limited to bacteria, and very little is known on how eukaryote microbes fit in the overall microbial ecology of the human gut.

## PERSPECTIVE

Humans coevolved alongside a multikingdom gut microbial ecosystem that includes microbial eukaryotes (microeukaryotes) among ancestral inhabitants of the human gut (1). The gut microbial eukaryome groups all nucleated life forms, including metazoan parasites (cestodes, nematodes, helminths, etc.), fungi (i.e., filamentous fungi and yeasts), and protozoans (2). For the purpose of this article, we have focused our discussion on protozoan and fungal organisms.

Industrial and scientific advances in the last two centuries have impacted human colonization with microbial eukaryotes in several ways. The routes of dispersion of many protozoans have drastically changed through the improvement of sanitation practices (access to potable water, “deworming” campaigns, improved personal hygiene, enhanced food sanitation, etc.) (3). These new dispersal barriers have created a colonization deficit in the gut microbiome of industrialized populations, a process that remains poorly studied in the context of microbiome ecology and the development of immune-mediated diseases. Simultaneously, the fungal microbiome, also known as the “mycobiome,” has been impacted by the surge in antibiotic use in animal and human populations. Antibiotic-induced bacterial modifications lead to drastic changes in bacterial metabolic output, which can strongly alter fungal colonization, survival, and infectious potential (see reference 4 for a review). Members of the gut mycobiome, including the ubiquitous yeast *Candida albicans*, are known to elicit strong immune responses (5). However, studies on antibiotic-induced changes to the microbiome rarely delve into nonbacterial microbes.

Microeukaryotes have been traditionally studied for their parasitic (protozoans) or pathogenic (fungi) relationships with the host, although they are increasingly being recognized to include commensal and potentially beneficial members of the gut microbiome (1). Here, we discuss some of the studies describing novel associations between specific microeukaryotes and gut bacterial ecology, as well as with immune diseases known to be influenced by the gut microbiome. As previously described in aquatic microbial ecosystems (6), the presence of key protozoans in the human gut has been associated with a substantial increase in bacterial diversity (7) and a change in community composition (8). In this context, we discuss the role of certain protozoan species as ecosystem engineers—organisms with the capacity to act as habitat builders, transformers, preservers, and destroyers (9) with strong consequences on co-occurring species fitness. As such, we hypothesize that the loss of key microeukaryotes can result in a domino effect on the gut bacterial community and in turn on host immunity, thus contributing to the differences observed between industrialized and nonindustrialized populations and the upsurge in immune-mediated diseases.

In light of the ecological and disease-related evidence discussed here, we emphasize the imperative need to include microeukaryotes in all future human gut microbiome studies and to prioritize the characterization of specific bacterial-eukaryotic interactions in the gut.

## ECOLOGICAL CONSIDERATIONS

Taxonomic surveys of the human gut eukaryome carried out thus far revealed that microbial eukaryotes are ubiquitously present in fecal samples, albeit at a much lower species richness and abundance and at a higher interindividual variability than bacteria (10–13). A recent amplicon-based analysis of the internal transcribed spacer region 2 (ITS-2) gene of 317 fecal samples of the Human Microbiome Project (13) revealed that fungal taxa were detectable in over 98% of samples, with the fungi *Saccharomyces*, *Candida*, and *Malassezia* as the most common genera in healthy North Americans. Our own studies in 250 healthy rural Mexicans, using 18S as a marker gene, found *Saccharomyces*, *Pichia*, and *Aspergillus* as the most abundant genera (unpublished observations). This finding suggests that, just like the fecal bacterial community, the fungal composition differs between sociogeographical settings. Compositional differences can also be detected during early infancy, as revealed by comparing an early-life microbiome study in North American babies by Fujimura et al. (14) with our recent analysis of the infant microbiome of Ecuadorian infants (15). Notably, the study by Fujimura et al. suggests that fungal species are present at higher richness in the first months of life compared to later months (14). This study also showed that the change in fungal diversity inversely correlates with bacterial diversity (14), suggesting that early-life ecological patterns of microbial alpha diversity involve fungal-bacterial interactions. It remains unknown how this reciprocal association occurs, yet it is likely that it involves (i) specific fungal-bacterial agonistic or antagonistic interactions and/or (ii) bacterial metabolism limiting fungal diversity during the first months of gut microbiome establishment.

There is a strong body of evidence on fungal-bacterial cellular and metabolic interactions. One well-studied example is the reciprocal influence of *Pseudomonas aeruginosa* with *Candida albicans*, two common residents of the lung microbiome associated with cystic fibrosis (16). *P. aeruginosa* can inhibit the filamentation of fungal cells, a transformation associated with *C. albicans* virulence (17, 18). In turn, *C. albicans* has the ability to inhibit the swarming motility of *P. aeruginosa* (16). Furthermore, bacterial fermentative metabolism in the gut, through the production of short-chain fatty acids, inhibits the growth of *C. albicans* (19). Although other examples of similar interactions are known to occur in the oral and vaginal microbiomes (see reference 20 for a review), little is known about community-level interactions.

**FIG 1:**
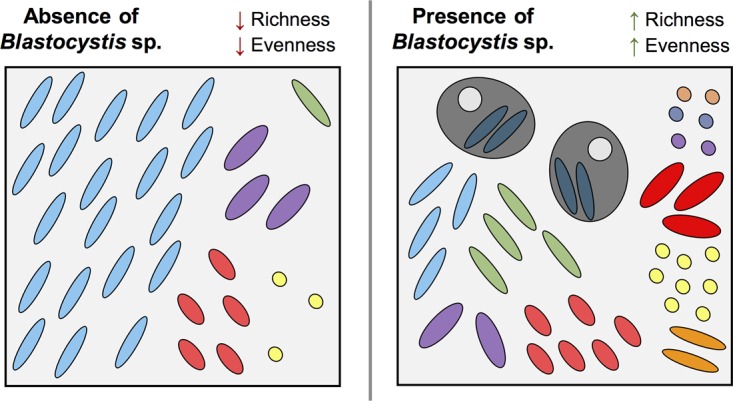
Proposed ecological role of *Blastocystis* sp. in the gut microbiota. In the absence of *Blastocystis* (left panel), a strong bacterial competitor dominates the community, which limits species richness and community evenness; when present (right panel), its predation on abundant bacterial taxa lowers the competition for nutrients and space, which leads to an increase in bacterial richness and community evenness. Without accounting for *Blastocystis* sp., the scenario in the right panel could be attributed to another variable.

Another emerging observation from recent eukaryome studies is the presence (or absence) of *Blastocystis* sp. This prevalent protozoan colonizes the intestine of approximately 0.5 to 30% of humans in industrialized countries and 30 to 100% in nonindustrialized societies (7, 8, 21–23). Its role in human health and disease is controversial as it is detected in healthy (8, 24–26) and diseased (27–29) individuals. Notably, the presence of this common protozoan is associated with a significant increase in bacterial alpha diversity (7) and with important compositional shifts in abundant bacterial taxa (8). We made similar observations in healthy individuals of rural Mexico (unpublished), suggesting that these changes are not a result of intestinal inflammation and may be due to interkingdom interactions between this protozoan and the bacterial community. Food web theory suggests that the increase in community diversity through grazing or predation can be explained by a top-down control on the strongest competitors, which consequently allows for the colonization and persistence of weaker competitors in the community; a dynamic supported by examples from both macroecology (30) and microecology (31). Although much remains unanswered as to how *Blastocystis* interacts with other members of the gut microbiome, Dunn et al. (32) reported that the ameboid form of the protozoan was able of bacterial engulfment, a process that has been suggested to serve the nutritious need of encystation (33). Future studies should test the hypothesis that *Blastocystis* sp. has a predatory effect on the gut microbial community, which leads to a general increase in alpha diversity (Fig. 1).

Although much remains to be elucidated on how microeukaryotes can support bacterial diversity, multitrophic network stability has been shown to be highly dependent on the size ratio between trophic levels (34). As potential ecosystem engineers, the loss of larger-sized predator organisms such as *Blastocystis* sp. likely exerts a significant impact on smaller prey organisms such as bacteria.

## INFLUENCE ON IMMUNE DISEASE

Gut microbes are implicated in the pathogenesis of immune-mediated diseases, such as inflammatory bowel disease (IBD) and asthma, in which a causal association has been confirmed in relevant models of disease (35, 36). Until recently, all of these studies had only detected bacteria, but recent reports also implicate gut fungi.

Microbiome studies in pediatric (37) and adult (38, 39) Crohn’s disease (CD) patients revealed marked fungal dysbiosis in association with disease. Notably, the abundance of a *Candida* species was significantly higher in CD individuals than in healthy first-degree relatives and positively correlated with levels of anti-*Saccharomyces* antibodies (ASCA) (38), an antibody marker commonly used in IBD diagnosis. One of these studies identified consistent correlations between bacterial and fungal taxa specific to IBD subjects, suggesting the existence of interkingdom alterations associated with this disease (39). Studies in mice revealed that prolonged treatment with the antifungal fluconazole, leading to fungal expansion of opportunistic species *Aspergillus amstelodami*, *Epicoccum nigrum*, and *Wallemia sebi*, worsened dextran sulfate sodium (DSS)-induced and T-cell transfer-mediated colitis (40).

Microbiome studies in prospective infant cohorts have also revealed gut fungal alterations preceding asthma development. We recently discovered marked fungal dysbiosis in 3-month-old Ecuadorian babies associated with subsequent asthma risk. In this study (15), as well as in a recent United States infant study (14), atopy was more strongly associated with fungal dysbiosis than with bacterial dysbiosis (15). Notably, in the Ecuadorian study, overrepresentation of fungal sequences is driven by significant increases in certain fungal taxa in babies at risk of asthma (15), as well as significant positive correlations between antibiotic use and fungal taxon abundance, suggesting a potential role of antibiotic-induced fungal overgrowth during early infancy in asthma development.

While previously overlooked, the mycobiome is altered after antibiotic treatment (41), a risk factor for asthma and other diseases. Until recently, we and others had limited our focus to antibiotic-driven bacterial dysbiosis in an attempt to elucidate potential causes for these changes, yet our recent multikingdom microbiome study also suggests that antibiotic use impacts the mycobiome. Being well-established that certain fungi have strong immunomodulatory effects (42), future studies should explore the immune consequences of both fungal dysbiosis and blooms during early life. Such studies are challenging as they must disentangle the immune effects caused by bacteria, but the use of gnotobiotic disease models makes these experiments possible.

## CONCLUDING REMARKS

As modern sequencing technology continues to increase the pace and depth of taxonomic and functional characterizations of the human gut microbiome, emergent studies have provided exciting new findings that involve microbial eukaryotes. A misconception of the overall importance of microeukaryotes is their reduced abundance in comparison to bacteria. However, the impact of a species on community structure and function is not necessarily proportional to its relative abundance. Macro- and microecology studies provide plenty of examples of the strong structuring consequences of cascading effects of low-abundance species, crossing multiple trophic levels and modulating the relative abundance of many species (43, 44). Current data support the role of *Blastocystis* sp. as a potential ecosystem engineer of the gut microbiome, likely capable of influencing the overall microbiome structure, as well as interacting and modulating the host immune system. Future ecological experiments should test this for *Blastocystis* sp., as well as other protozoan species more commonly found in nonwesternized settings.

Given the evidence discussed here, we propose that, just like current microbiome studies must account for the effects of diet, age, antibiotics, inflammation, and other variables known to influence the microbial community, new human microbiome studies should integrate and account for the effect of microbial eukaryotes. Finally, given the crucial interrelationship between the infant gut microbiome and many host physiological developmental processes, it is of utmost importance to understand how early-life patterns of diversity occur, how they are influenced by interkingdom interactions, and how these community patterns influence infant development. This will provide a more integrated view on gut microbiomes, the factors that shape them, and the mechanisms by which they relate to health and disease.
